# Fenfluramine: a plethora of mechanisms?

**DOI:** 10.3389/fphar.2023.1192022

**Published:** 2023-05-12

**Authors:** Jo Sourbron, Lieven Lagae

**Affiliations:** Department of Development and Regeneration, Section Pediatric Neurology, University Hospital KU Leuven, Leuven, Belgium

**Keywords:** fintepla, pathways, serotonin, sigma, disease modification, epilepsy

## Abstract

Developmental and epileptic encephalopathies are rare, treatment-resistant epilepsies with high seizure burden and non-seizure comorbidities. The antiseizure medication (ASM) fenfluramine is an effective treatment for reducing seizure frequency, ameliorating comorbidities, and potentially reducing risk of sudden unexpected death in epilepsy (SUDEP) in patients with Dravet syndrome and Lennox-Gastaut syndrome, among other rare epilepsies. Fenfluramine has a unique mechanism of action (MOA) among ASMs. Its primary MOA is currently described as dual-action sigma-1 receptor and serotonergic activity; however, other mechanisms may be involved. Here, we conduct an extensive review of the literature to identify all previously described mechanisms for fenfluramine. We also consider how these mechanisms may play a role in the reports of clinical benefit in non-seizure outcomes, including SUDEP and everyday executive function. Our review highlights the importance of serotonin and sigma-1 receptor mechanisms in maintaining a balance between excitatory (glutamatergic) and inhibitory (γ-aminobutyric acid [GABA]-ergic) neural networks, and suggests that these mechanisms may represent primary pharmacological MOAs in seizures, non-seizure comorbidities, and SUDEP. We also describe ancillary roles for GABA neurotransmission, noradrenergic neurotransmission, and the endocrine system (especially such progesterone derivatives as neuroactive steroids). Dopaminergic activity underlies appetite reduction, a common side effect with fenfluramine treatment, but any involvement in seizure reduction remains speculative. Further research is underway to evaluate promising new biological pathways for fenfluramine. A better understanding of the pharmacological mechanisms for fenfluramine in reducing seizure burden and non-seizure comorbidities may allow for rational drug design and/or improved clinical decision-making when prescribing multi-ASM regimens.

## 1 Introduction

Epilepsy, a neurological disorder characterized by seizures, affects up to 70 million people worldwide (Singh and Trevick, 2016). The mainstay of treatment remains controlling seizures by antiseizure medications (ASMs). Since epilepsy is a heterogeneous condition, there is no perfect ASM for all epilepsy patients. The optimal treatment strategy is dependent on etiology, patient-specific factors (e.g., seizure type, sex, age, comorbidities, family history) and ASM characteristics (drug interaction profile, adverse effects, costs) (www.nice.org.uk/guidance/CG137). ASMs can act through different pathways and subsequently increase neuronal inhibition and/or decrease neuronal excitation. A primary mechanism of many ASMs is by sodium channel blockade and/or enhancement of neurotransmission by *γ*-aminobutyric acid (GABA) (Löscher and Klein, 2021; Strzelczyk and Schubert-Bast, 2022). The development of ASMs increased tremendously in the past 30 years. Second- and third-generation ASMs have various novel molecular targets (e.g., voltage-gated cation channels, glutamate [GLUT], GABA turnover, synaptic vesicle protein 2A) (Loscher et al., 2013; Löscher, 2021).

Nonetheless, about one-third of patients with epilepsy are unable to achieve seizure control on their current ASM regimens (Loscher et al., 2013) (i.e., patients with developmental and epileptic encephalopathies [DEE]). Patients with DEE experience severe, drug-resistant seizures and developmental delay due to both epileptiform activity and the underlying pathology of their condition (Specchio et al., 2022). DEE can cause developmental, social, emotional, and physical dysfunctions secondary to seizures or as a direct result of either the underlying pathology or the induced neurochemical alterations (Nabbout et al., 2013; Gataullina and Dulac, 2017). To reduce seizure frequency and, ideally, also alleviate comorbidities, ASMs should have novel, preferably multimodal, mechanisms of action.

In this short review, we will focus on fenfluramine (FFA), an ASM with mechanisms of action unique among ASMs (Reeder et al., 2021a; Martin et al., 2021), which is now approved in the US, Europe, the UK, and Japan as add-on therapy in patients with Dravet syndrome, as well as in the US for treating patients with Lennox-Gastaut syndrome (LGS) (Zogenix, 2022). Clinical trials are currently underway to evaluate FFA’s potential as an ASM for other DEEs when added to a patient’s current standard-of-care regimen (https://clinicaltrials.gov/ct2/show/NCT05232630) (Devinsky et al., 2021; Aledo-Serrano ÁCabal-Paz et al., 2022).

FFA’s mechanisms of action have been studied extensively. High-dose FFA (60–120 mg/day) was originally marketed as an anti-obesity drug that reduced food intake through serotonergic activation of hypothalamic energy homeostasis circuits. With the discovery of its potent antiseizure properties (Schoonjans et al., 2015), low-dose FFA (0.2–0.7 mg/kg/day; maximum 26 mg/day) was re-developed to an ASM (Schoonjans et al., 2015; Johannessen Landmark et al., 2021). The pharmacological mechanisms underlying the antiseizure effects of FFA have been the subject of extensive research in recent years. According to the current hypothetical model, FFA enhances GABAergic signaling *via* activity at serotonin (5-hydroxytryptophan, 5-HT) receptors and inhibits excitatory signaling through sigma-1 (σ1)-mediated mechanisms, thereby restoring the balance between inhibition and excitation (Sourbron et al., 2017; Martin et al., 2020; Sourbron and Lagae, 2022). Nonetheless, other mechanisms are likely to be involved. Recent data suggest that FFA confers clinical benefit beyond seizure reduction alone (Jensen et al., 2022; Jensen et al., 2023), including improvements in everyday executive function (defined as self-regulation of emotions, behavior, and cognition or working memory operations) (Bishop et al., 2021a; Bishop et al., 2022a) and reduction in sudden unexpected death in epilepsy (SUDEP) (Cross et al., 2021).

Since there is currently no clear, comprehensive overview regarding FFA’s pharmacological mechanisms in the literature, our aim was to concisely summarize all the known mechanisms of FFA on seizures and ancillary mechanisms that may be related to seizure control, as well as consider additional mechanisms of its observed clinical benefit in non-seizure comorbidities and survival. Our PubMed search (July 2022) retrieved 622 articles, of which 79 contained relevant information regarding the mechanisms of FFA.

**FIGURE 1:**
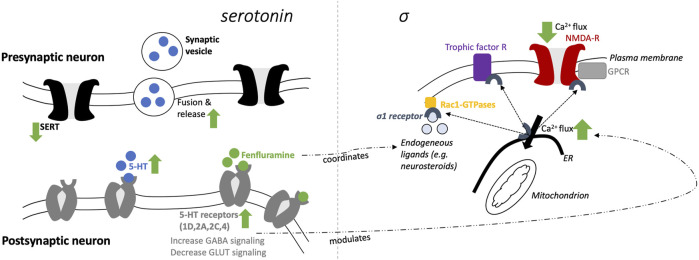
Schematic mechanisms of fenfluramine at a synaptic level (5-HT; left) and cellular level (σ1; right).

**FIGURE 2:**
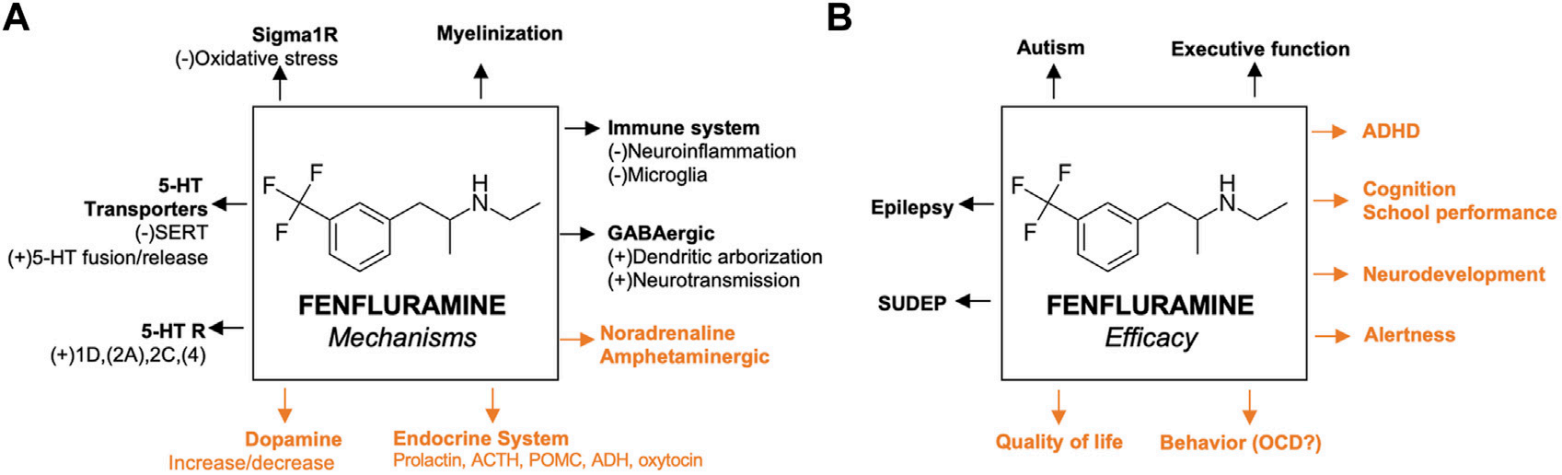
Proposed mechanisms of fenfluramine and its efficacy **(A)** Previously reported pathways are in black; mechanisms that are not yet confirmed in animal models of epilepsy are presented in orange **(B)** Efficacies confirmed by clinical data before the start of fenfluramine in clinical trials of epilepsy patients are presented in black; activities that were recently confirmed by clinical trials of epilepsy patients are presented in orange. For the corresponding references, please refer to the text. 5-HT, serotonin; ACTH, adrenocorticotropic hormone; ADHD, attention deficit hyperactivity disorder; GABA, *γ*-aminobutyric acid; GLUT, glutamine; OCD, obsessive compulsive disorder; σ1, sigma-1; SERT, serotonin transporter; SUDEP, sudden unexpected death in epilepsy.

The proposed mechanisms of fenfluramine at the synaptic level of neurotransmission in the context of DEEs are presented in Figure 1. At a synaptic and cellular level, FFA modulates serotonergic and σ1-related pathways, respectively (Figure 1, left and right, respectively). In Figure 2, we provide an overview of the A) mechanisms and B) clinical efficacy data of FFA.

## 2 Primary mechanisms of fenfluramine antiseizure activity

### 2.1 Serotonergic neurotransmission

Fenfluramine (FFA, 3-trifluoromethyl-N-ethylamphetamine) is a racemic mixture of levo-FFA and dextro-FFA (Balagura et al., 2020; Odi et al., 2021). Both enantiomers are rapidly metabolized to norfenfluramine, which is also pharmacologically active *via* multiple mechanisms (Marchant et al., 1992; Bever and Perry, 1997). Dextro-FFA (dexfenfluramine) promotes serotonergic neurotransmission by inhibition of serotonin (5-hydroxytryptophan, 5-HT) reuptake and stimulation of 5-HT release (Kannengiesser et al., 1976; Garattini and Samanin, 1978; Baumann et al., 2014). Subsequently, different 5-HT subtype receptors can be activated, of which several have been associated with the anticonvulsant effects of FFA in the last 5 years (Supplemental Table S1). In addition, FFA has agonist activity at distinct 5-HT receptors (see section on 5-HT receptors below). Furthermore, 5-HT itself plays a crucial role in normal brain physiology, and distinct 5-HT receptors are involved in seizure-reducing effects (as well as non-seizure outcomes). Hence, it is not surprising that defective serotonergic neurotransmission could be related to epilepsy (Di Giovanni, 2013; Guiard and Giovanni, 2015; Svob Strac et al., 2016; Zarcone and Corbetta, 2017; Deidda et al., 2021).

### 2.2 Serotonin receptors

Of the 14 known 5-HT receptors, six subtypes have confirmed FFA activity (Supplemental Table S1), including agonist activity at 5-HT1D, 5-HT2A, 5-HT2B, 5-HT2C, and 5-HT4, and antagonist activity at 5-HT1A (Rothman et al, 2003; Sourbron et al., 2017; Rodríguez-Muñoz et al., 2018; Tupal and Faingold, 2019; Martin et al., 2020; Reeder et al., 2021b; Tupal and Faingold, 2021). Activity at 5-HT7 has more recently been described (Faingold and Tupal, 2019). Early binding studies showed high affinity of norfenfluramine for 5-HT2A, 5-HT2B, and 5-HT2C, while fenfluramine was a weak agonist with low affinity for any 5-HT2 receptor (Porter et al., 1999; Rothman et al., 2003). Binding assays confirmed low affinity for 5-HT1A, with antagonist activity in functional assays *in vitro* (Martin et al., 2020). Studies in a zebrafish model of Dravet syndrome demonstrated that treatment with FFA in the presence of antagonists to 5-HT1D, 5-HT2A, and/or 5-HT2C receptors no longer inhibited spontaneous seizures, suggesting that agonist activity at these receptor subtypes may be responsible for reducing seizure frequency (Sourbron et al., 2017). Consistent with this report, subsequent studies showed that other 5-HT1D agonists were effective in several zebrafish seizure models (Sourbron et al., 2016; Sourbron et al., 2017; Gooshe et al., 2018; Sourbron et al., 2019) and two rodent seizure models (Gooshe et al., 2018; Hatini and Commons, 2020). More recent preclinical studies showed that seizure reduction and/or reduction of SUDEP by FFA also may be associated, at least partially, with stimulation of the 5-HT4 (Tupal and Faingold, 2021).

FFA was reported to induce valvular heart disease and pulmonary arterial hypertension, potentially due to 5-HT2B stimulation (Rothman et al., 2000); however, these effects were most likely related to high dosages (up to 160 mg/day), combination treatment with other 5-HT2B agonists (such as phentermine), and/or other cardiovascular risk factors (older age/female sex/hypertension) (Rothman et al., 2000). The 5-HT2B receptor subtype is expressed in low abundance in the brain (Rothman et al., 2000; Launay et al., 2002; Elangbam, 2010; Sourbron and Lagae, 2022) and does not appear to play a role in FFA’s antiseizure effects (Sourbron et al., 2017; Odi et al., 2021). Current long-term clinical data support the cardiovascular safety of FFA in treating patients with epilepsy at much lower dosages (Schoonjans and Ceulemans, 2022). In a comprehensive long-term open label study conducted in patients with Dravet syndrome, no cases of valvular heart disease or pulmonary arterial hypertension were reported in 327 patients treated with FFA for a median treatment duration of 23.9 months at a median FFA dose of 0.44 mg/kg/day (Agarwal et al., 2022). Regular follow-up echocardiography is advised before initiating FFA and during treatment (Schoonjans et al., 2017).

As summarized in a prior review (Sourbron and Lagae, 2022), the serotonergic mechanisms of FFA include: 1) increase of GABAergic dendritic arborization *via* serotonergic and GABAergic activity (see below); 2) decrease of 5-HT reuptake by inhibition of 5-HT transporters (SERT); 3) increase of release and fusion of synaptic vesicles (filled with 5-HT); 4) 5-HT increase in the synaptic cleft—via (2) and (3)—and subsequently stimulation of different 5-HT receptor subtypes and 5) direct stimulation of at least four.

5-HT receptor subtypes (5-HT1D, 2A, 2C, and 4), which increases GABA inhibitory input and decreases glutaminergic excitatory output.

### 2.3 Sigma-1 pathway

FFA has high (sub-micromolar) affinity for the σ1 receptor (Martin et al., 2020). Contradictory findings have been reported about the action of FFA on σ1 receptors (Rodríguez-Muñoz et al., 2018; Sourbron and Lagae, 2022). However, a growing body of corroborating evidence supports that FFA acts as a positive modulator of σ1 receptors. In a mouse model of dizocilpine-induced learning deficits, FFA acted as a positive modulator of σ1 receptors (Martin et al., 2020). Further, dextro-FFA reduced dizocilpine-induced deficits in spatial memory by positive modulation of 5-HT receptors by the σ1 receptor (Martin et al., 2022). Further *in vitro* and *in vivo* studies underlined these positive modulatory effects of FFA, which were related to their antiseizure activities (Vavers et al., 2019; Martin et al., 2021) and potentially also contribute to the prevention of SUDEP (Ning et al., 2021). The reason for both agonist and antagonist activity reported at the σ1 receptor with FFA treatment is unclear, but may be due to the biphasic dose response of σ1 receptor modulation (Maurice, 2021).

As outlined concisely in a prior review (Martin et al., 2021), FFA restores the loss of GABAergic tone *via* mediating the σ1 interaction with the NMDA receptor that leads to a dampening of calcium influx and decreasing seizure activities at glutaminergic synapses. Modulation of these calcium fluxes (in the endoplasmic reticulum *via* a Gq/inositolphosphate3-receptor mediated mechanism) is also under the control of serotonergic neurotransmission. σ1 receptor-client protein interactions initiate a host of signal transduction cascades, including other ion channels besides NMDA (e.g., potassium, sodium, and voltage-regulated chloride channels), as well as interaction with trophic factor receptors and kinases. Finally, the interaction with Rac-GTPases promotes dendritic spine formation and affects neuronal redox processes, which likely contributes to its antiseizure (Vavers et al., 2017) and potentially antidepressant effects (Voronin et al., 2020). The effects of FFA on these downstream second messenger systems remain to be elucidated, but FFA interaction with the σ1 receptor could potentially mediate any of these downstream effects to produce antiseizure effects. Further studies are needed to determine which second messenger systems contribute to antiseizure effects of FFA in response to σ1 receptor activation.

### 2.4 GABA neurotransmission

The loss of GABAergic neurotransmission is a major contributor to epileptogenesis in numerous preclinical models of epilepsy (de Lanerolle et al., 1989; Sundstrom et al., 2001; Swartz et al., 2006; Oakley et al., 2011; Houser, 2014). FFA enhances GABAergic neurotransmission by 5-HT release at GABAergic synapses and stimulating 5-HT2A and 5-HT2C receptors (Shen and Andrade, 1998; Higgins et al., 2014; Martin et al., 2014; Guiard and Giovanni, 2015). Further, FFA has been shown to restore dendritic arborization of GABAergic neurons in a Dravet syndrome zebrafish model of Dravet syndrome (Tiraboschi et al., 2020). Taken together, FFA may restore inhibitory synaptic inputs by a combined effect of preserving the GABAergic dendritic architecture and enhancing GABA neurotransmission in Dravet syndrome and other DEEs.

## 3 Ancillary mechanisms of fenfluramine

### 3.1 Dopaminergic neurotransmission

Levo-FFA, lacking serotonergic activity in contrast to dextro-FFA, can modulate dopaminergic transmission (Invernizzi et al., 1989; Baumann et al., 2000; Wurtman and Fenfluramine, 2018). Some studies reported that the increase of extracellular dopamine by FFA is mediated by its primary effect on 5-HT (Balcioglu and Wurtman, 1998; Ledonne et al., 2009). However, these effects on dopaminergic transmission are rather small compared to 5-HT modulation (Crespi et al., 1997; Rothman et al., 2008) and appear to be high-dose related (Balcioglu and Wurtman, 1998). Furthermore, FFA does not seem to bind directly to dopaminergic receptors (Invernizzi et al., 1989; Martin et al., 2020). There is only one case report that links the assumed dopaminergic-enhancing effects of FFA to seizure control (Clemens, 1988), and moreover this study did not involve experiments to prove that the beneficial effects of FFA on self-induced seizures were related to dopamine. In contrast, other studies suggest a decrease of dopamine or dopaminergic neurotransmission by FFA (Garattini and Samanin, 1978; Invernizzi et al., 1989; Sourbron et al., 2017). The impact of FFA on dopaminergic modulation appears to be more relevant to reduced appetite (a known side effect of FFA) than to seizure control by affecting the pleasurable aspects of feeding behavior (Rothman et al., 2008; Ledonne et al., 2009).

### 3.2 Noradrenergic neurotransmission

FFA modulates noradrenergic neurotransmission, a mechanism that may contribute to the clinical benefit associated with amelioration of concentration problems, learning difficulties, and attention deficit hyperactivity disorder (ADHD) (Donnelly et al., 1989; Aman et al., 1993; Reeder et al., 2021a; Jensen et al., 2021; Jensen et al., 2022). However, in epilepsy patients, FFA-associated improvements in cognitive domains, including self-regulation and everyday executive function, can also be related to FFA-induced seizure reduction (Besag and Vasey, 2021) in addition to a direct effect not mediated by seizure reduction (Martin et al., 2022).

FFA has direct effects on adrenergic receptors and their target receptors. At high, supratherapeutic concentrations *in vitro* (>10 µM), dextro-FFA can stimulate alpha 1-adrenergic receptors, resulting in a metabolic shift from glucose production (gluconeogenesis) to glycose degradation (glycolysis), which is mediated by a change in glucose 6-phosphate (Comte et al., 1997). One could speculate that the increase of glycolysis could be related to a decrease in epileptic activity since decreased glycolysis impairs neuronal function, and glycolysis sustains normal synaptic function (Li et al., 2000). However, most studies indicate that inhibition, rather than stimulation, of glycolysis is associated with antiseizure activities (Fei et al., 2020). Without additional data with FFA at physiologically relevant concentrations, it is difficult to conclude what effects FFA has on alpha-adrenergic receptors, and whether these effects contribute to FFA’s antiseizure effects observed in patients with DEEs.

Inhibiting beta-adrenergic receptors attenuated maximal electroshock-induced seizures in mice and audiogenic seizures in DBA/2 mice (Lints and Nyquist-Battie, 1985; Luchowska et al., 2001), suggesting a role for beta-adrenergic receptors in epileptogenesis. FFA and norfenfluramine bind to beta 2-adrenergic receptors with micromolar affinity (1.26 x 10^−5^ and 8.77 x 10^−6^, respectively (Martin et al., 2016)). Selective antagonism of beta 2-adrenergic receptors in a zebrafish model of Dravet syndrome had no effect on spontaneous epileptiform activity (Sourbron et al., 2017), arguing against a direct effect on this receptor subtype. However, we cannot exclude an indirect effect of FFA on the adrenergic receptors and their pharmacologic targets, as FFA can decrease the noradrenaline content in the brains of zebrafish larvae (Sourbron et al., 2017) and rats (Calderini et al., 1975). This decrease is likely the result of FFA’s effects on 5-HT (Astorne Figari et al., 2014). Of interest, elevated noradrenaline transmission has been related to some cases of epilepsy (Fitzgerald, 2010) and even though there are contradictory data (Svob Strac et al., 2016), there clearly is evidence for the use of noradrenaline-decreasing drugs for treating neurological diseases, including epilepsy (Fitzgerald, 2015). Taken together, the data suggest that any antiseizure activity of FFA on noradrenergic neurotransmission is likely to be an indirect result decreased levels of noradrenaline in the brain.

### 3.3 Endocrine system

FFA targets several neuropeptides, even though the exact role of these neuro-endocrinological activities remains elusive. First, FFA increases prolactin in humans (Kavoussi et al., 1999) and primates (Bethea et al., 2013). Although epileptiform activity in the hypothalamic pituitary axis (HPA) putatively causes prolactin secretion (Lusić et al., 1999), there is currently no evidence to support an antiseizure effect of prolactin secretion.

Second, 5-HT release by FFA stimulates 5-HT2C receptors in proopiomelanocortin (POMC) neurons of the hypothalamic melanocortin system that regulate energy homeostasis and feeding (Smith et al., 2010; He et al., 2021). The melanocortin peptide adrenocorticotropic hormone (ACTH) is another POMC derivative that is elevated after FFA treatment (Schürmeyer et al., 1996). These effects of FFA on the HPA axis have typically been interpreted in relation to the anorectic properties of FFA as a former anti-obesity drug. However, ACTH also has antiseizure activity and is commonly used as an ASM in treating DEEs such as LGS, and Ohtahara and West syndromes (Strzelczyk and Schubert-Bast, 2022). Further evidence is needed to determine whether activity of FFA on the HPA after 5-HT2C-induced ACTH release from POMC neurons contributes to its antiseizure activity.

Third, dextro-FFA specifically activates oxytocinergic and vasopressinergic neurons in the rat brain (Mikkelsen et al., 1999). The balance between oxytocin and vasopressin regulates emotions and behaviors such as anxiety and social behavior. Oxytocin also reduces epileptic seizures in preclinical studies (Erfanparast et al., 2017), and vasopressin is related to the pathogenesis of some epilepsies (Gulec and Noyan, 2002). Additional studies are needed to determine whether FFA affects the balance between oxytocin and vasopressin in a way that is clinically meaningful to its antiseizure effects.

Fourth, Martin et al. (2022) demonstrated that positive modulation of FFA and the dextro-FFA enantiomer (but not the levo-enantiomer) on σ1 receptors reversed dizocilpine-induced amnesia in rodent models, while norfenfluramine (both dextro- and levo-isomers) acted as an antagonist at σ1 receptors (Martin et al., 2022). Furthermore, FFA and dextro-FFA activity interacted synergistically with the neuroactive steroids pregnenolone sulfate or dehydroepiandrosterone sulfate (both σ1 receptor agonists), and progesterone (a σ1 receptor antagonist) blocked the anti-amnesic effect of FFA. These data suggested that the anti-amnesic effects of FFA may be mediated by amplification of endogenous σ1 receptor agonists such as neuroactive steroids. Antagonists to 5-HT1A and 5-HT2A inhibited the effects of FFA, suggesting that the interaction between σ1 receptors and neuroactive steroids may involve these receptor subtypes. Clinical studies with the neuroactive steroid ganaxolone suggest neuroactive steroids may have antiseizure efficacy in patients with DEEs by acting as non-competitive antagonists of GABA-A receptors (Knight et al., 2022), but further studies are needed to determine whether neuroactive steroids play a role in FFA’s effects on seizures.

In summary, data to date suggest that the effects of FFA on hormones associated with the HPA (e.g., ACTH, prolactin) or oxytocin/vasopressin are most likely to affect food intake, with only weak or speculative evidence for involvement in antiseizure properties. Neuroactive steroids (e.g., progesterone derivatives) are attractive candidates for further investigation.

## 4 Efficacy of fenfluramine, beyond seizures

Clinical and preclinical data support that FFA treatment may positively impact non-seizure comorbidities in addition to improving seizure control in patients with DEEs (Figure 2). First, FFA promoted survival in clinical data and preclinical models (Reeder et al., 2021b; Cross et al., 2021; Ning et al., 2021; Tupal and Faingold, 2021). FFA reduced SUDEP mortality rates compared to pre-treatment rates (1.7 deaths per patient-years after FFA compared to 11.7 deaths per patient-years pre-FFA treatment) and historical controls without FFA treatment (9.3 deaths per 1,000 person-years) (Cross et al., 2021). The mechanisms of these effects are under investigation, but some evidence supports a role for 5-HT4 and σ1 receptors (Ning et al., 2021; Tupal and Faingold, 2021). Preclinical data demonstrated that FFA reduced seizure-induced respiratory arrest in a mouse model of SUDEP by acting at 5-HT4 receptors (Tupal and Faingold, 2021). Additional preliminary data in a mouse model of Dravet syndrome showed that FFA reduced mortality of FFA-treated animals (Reeder et al., 2021b). This report showed that FFA may also reduce neuroinflammation, demyelination, and apoptosis in the hippocampus, corpus callosum, and/or parietal cortex, contributing to survival (Reeder et al., 2021b), but further studies are needed to confirm these preliminary results and definitively link these observations to SUDEP or survival.

Second, FFA improved everyday executive functioning, including regulation of emotions and behavior, in some patients with Dravet syndrome (Bishop et al., 2021a; Bishop et al., 2022a) and LGS (Bishop et al., 2021c; Bishop et al., 2022b; Bishop et al., 2021b). These effects of FFA appeared to be, at least in part, independent of seizure control (Bishop et al., 2022a). The mechanism of these effects remains to be established, but data in a dizocilpine-induced amnesia model suggests that FFA improves spatial learning and memory (i.e., aspects of cognition) by positively modulating σ1 receptors (Martin et al., 2020). Further, the activity of FFA at 5-HT4 receptors may positively affect cognition, as evidence suggests that 5-HT4 receptor agonism enhances learning and memory in clinical studies (Murphy et al., 2020). Survey data of caregivers of patients with Dravet syndrome suggest additional clinical benefit beyond seizure control after FFA treatment, including improved cognitive function, alertness, education-related outcomes, and focus (Jensen et al., 2022; Jensen et al., 2023). Additional clinical and preclinical data suggest improvement of autistic-like behavior, obsessive-compulsive behavior, everyday executive functioning (i.e., self-regulation of emotions, cognition, and behavior), alertness, cognition, and QoL with FFA treatment (Gastaut, 1984; Aicardi and Gastaut, 1985; Donnelly et al., 1989; Hollander et al., 1992; Aman et al., 1993; Higgins and Fletcher, 2015; Schoonjans et al., 2017; Bishop et al., 2021a; Bishop et al., 2021c; Reeder et al., 2021b; Jensen et al., 2021; Bishop et al., 2022a; Bishop et al., 2022b; Jensen et al., 2022; Bishop et al., 2021b). Additional studies are needed to determine the mechanisms underlying these observations in relation to the clinical effects observed after FFA treatment in patients with DEEs.

## 5 Conclusion

Current therapeutic approaches to treating severe DEEs support targeting multiple mechanisms to optimize clinical efficacy. Rationally designed ASMs have targeted a single receptor or pathway (Roth et al., 2004). More recently, ASMs or combinations of ASMs with multimodal mechanisms of action have been developed to improve clinical efficacy in treating seizures and non-seizure comorbidities (Cardamone et al., 2013). FFA is an ASM with multimodal mechanisms of action. We consider dual-action 5-HT and σ1 receptor activity to be the primary pharmacological mechanisms of action for FFA’s antiseizure effects. Those mechanisms, which are well-supported by preclinical data on antiseizure effects, include balancing inhibitory (GABAergic) and excitatory (glutamatergic) inputs by: 1) serotonergic neurotransmission and 5-HT receptor activation, 2) enhancing GABAergic neurotransmission and preserving GABA neuron dendritic arborization, and 3) activity at the σ1 receptor. We also reviewed additional pharmacological mechanisms demonstrated in the literature for FFA and evaluated the strength of the evidence mediating antiseizure activity and comorbidities. Of the pathways described, some evidence exists for neuroactive (progesterone-derivative) steroids, with weaker or speculative evidence for ACTH, noradrenergic, or dopaminergic endocrine systems. Interesting additional speculative pharmacological pathways for further research include myelination and neuroinflammation. It is important to note that FFA’s multimodal mechanisms of action will not be mutually exclusive, but rather will act cooperatively in antiseizure and non-seizure effects. Overall, we delineate possible specific pathways relevant to FFA that may inform future studies and contribute to greater understanding of the pharmacological mechanisms of action of FFA in treating epilepsy and other conditions.
