# Supplementary material for: Fenfluramine: a plethora of mechanisms?
Source: Front Pharmacol. 2023 May 12;14:1192022. doi: 10.3389/fphar.2023.1192022 (PMC10213522; doi:10.3389/fphar.2023.1192022)
Supplement: Supplementary file 1 [file Table1.pdf]

Table S1. Cell-Based and In Vivo Pharmacology Studies Supporting Serotonergic Activity of Fenfluramine or Major Metabolite Norfenfluramine

Receptor (Reference)	FFA or NFA activity	Model(s)	G-protein coupling	Second messenger response to FFA or NFA	Predicted cellular response to FFA or NFA
5-HT1D (1)	Confirmed agonist	Cell-based pharmacology; DS mouse; MES mouse; DS zebrafish	Coupled to Gi/Go	Decreased cellular levels of cAMP	Inhibitory
5-HT2A (1-3)	Confirmed agonist	Cell-based pharmacology; MES mouse; DS zebrafish; SUDEP mouse[a]	Coupled to Gq/G11	Increased cellular levels of IP_3 and DAG	Excitatory
5-HT2B (4,7)	Confirmed agonist	Cell-based pharmacology[b]	Coupled to Gq/G11	Increased cellular levels of IP_3 and DAG	Excitatory
5-HT2C (1,4-6)	Confirmed agonist	Cell-based pharmacology; DS zebrafish	Coupled to Gq/G11	Increased cellular levels of IP_3 and DAG	Excitatory
5-HT4 (5,6)	Potential agonist	SUDEP mouse	Coupled to Gs	Increased cellular levels of cAMP	Excitatory
5-HT1A (2,3,5,6)	Potential antagonist	Cell-based pharmacology; SUDEP mouse	Coupled to Gi/Go	Increased cellular levels of cAMP	Excitatory
**Additional Serotonergic Mechanisms**					
**Target**	**Activity**	**Model(s)**	**Mechanism**		**Predicted cellular response**
5-HT releaser (8)	Increased 5-HT release into synaptic cleft	Cell-based assays; Xenopus oocytes In vivo rat	SERT substrate, reverse transporter		Excitatory

Cell-based pharmacology agonist assays found no activity at 5-HT1B, 5-HT1E, 5-HT1F, 5-HT3, 5-HT5A, 5-HT6, and 5-HT7.

[a]5-HT2 antagonist (ritanserin) enhanced FFA-mediated reduction in seizures and SUDEP; these data contradict cell-based pharmacology, MES mouse, and DS zebrafish in vivo data.

[b]5-HT2B was not active in DS zebrafish model.

DAG, diacylglycerol; DS, Dravet syndrome; FFA, fenfluramine; IP_3, inositol triphosphate; MES, maximal electroshock seizure model; NFA, norfenfluramine; SUDEP, sudden unexpected death in epilepsy.

1. Sourbron J, Smolders I, de Witte P, Lagae L. Pharmacological analysis of the anti-epileptic mechanisms of fenfluramine in scn1a mutant zebrafish. *Frontiers in Pharmacology* (2017) 8. doi: 10.3389/fphar.2017.00191
2. Martin P, de Witte PAM, Maurice T, Gammaitoni A, Farfel G, Galer B. Fenfluramine acts as a positive modulator of sigma-1 receptors. *Epilepsy & Behavior* (2020) 105:106989. doi: 10.1016/j.yebeh.2020.106989
3. Rodríguez-Muñoz M, Sánchez-Blázquez P, Garzón J. Fenfluramine diminishes NMDA receptor-mediated seizures via its mixed activity at serotonin 5HT2A and type 1 sigma receptors. *Oncotarget* (2018) 9:23373-89. doi: 10.18632/oncotarget.25169
4. Rothman RB, Clark RD, Partilla JS, Baumann MH. (+)-Fenfluramine and its major metabolite, (+)-norfenfluramine, are potent substrates for norepinephrine transporters. *J Pharmacol Exp Ther* (2003) 305:1191-9. doi: 10.1124/jpet.103.049684
5. Tupal S, Faingold CL. Fenfluramine, a serotonin-releasing drug, prevents seizure-induced respiratory arrest and is anticonvulsant in the DBA/1 mouse model of SUDEP. *Epilepsia* (2019) 60:485-94. doi: 10.1111/epi.14658
6. Tupal S, Faingold CL. Serotonin 5-HT4 receptors play a critical role in the action of fenfluramine to block seizure-induced sudden death in a mouse model of SUDEP. *Epilepsy Research* (2021) 177. doi: https://doi.org/10.1016/j.eplepsyres.2021.106777
7. Tupal S, Faingold CL. Serotonin 5-HT4 receptors play a critical role in the action of fenfluramine to block seizure-induced sudden death in a mouse model of SUDEP. *Epilepsy Res* (2021) 177:106777. doi: https://doi.org/10.1016/j.eplepsyres.2021.106777
8. Baumann MH, Bulling S, Benaderet TS, Saha K, Ayestas MA, Partilla JS, et al. Evidence for a role of transporter-mediated currents in the depletion of brain serotonin induced by serotonin transporter substrates. *Neuropsychopharmacology* (2014) 39:1355-65. doi: 10.1038/npp.2013.331
